# The Effect of Stress on Motor Function in *Drosophila*

**DOI:** 10.1371/journal.pone.0112076

**Published:** 2014-11-06

**Authors:** Abhishek Chadha, Boaz Cook

**Affiliations:** Department of Molecular and Cellular Neuroscience, The Scripps Research Institute, La Jolla, California, United States of America; Alexander Fleming Biomedical Sciences Research Center, Greece

## Abstract

Exposure to unpredictable and uncontrollable conditions causes animals to perceive stress and change their behavior. It is unclear how the perception of stress modifies the motor components of behavior and which molecular pathways affect the behavioral change. In order to understand how stress affects motor function, we developed an experimental platform that quantifies walking motions in *Drosophila*. We found that stress induction using electrical shock results in backwards motions of the forelegs at the end of walking strides. These leg retrogressions persisted during repeated stimulation, although they habituated substantially. The motions also continued for several strides after the end of the shock, indicating that stress induces a behavioral aftereffect. Such aftereffect could also be induced by restricting the motion of the flies via wing suspension. Further, the long-term effects could be amplified by combining either immobilization or electric shock with additional stressors. Thus, retrogression is a lingering form of response to a broad range of stressful conditions, which cause the fly to search for a foothold when it faces extreme and unexpected challenges. Mutants in the cAMP signaling pathway enhanced the stress response, indicating that this pathway regulates the behavioral response to stress. Our findings identify the effect of stress on a specific motor component of behavior and define the role of cAMP signaling in this stress response.

## Introduction

Animals adjust their motions according to external conditions. Normally, perturbations such as forces opposing motion [1] or impediments blocking stride execution [2] cause animals to adapt to external conditions. When perturbations are unpredictable and uncontrollable, however, animals modify their behavior in a manner that does not necessarily help them to negotiate the perturbation [3]. Such modifications have been observed in a range of animals [4]–[8]. In the face of threats animals modify behaviors such as hiding [7], exploration behavior [9] and motion speed [5]. However, information about the effect of stress on the motions that comprise behavior are scarce [10].

Since motor adaptations and stress responses are distinct processes they manifest different features in response to prolonged perturbations. Motor adaptations to a sustained external perturbation manifest as a learning process, in which motions gradually change until they reach a modified state [1]. Conversely, the initial response to stress is maximal and repetition of stimulus results in a gradual decrease of responses due to habituation [11]. Thus, prolonged treatments that induce motor adaptation elicit a shift farther from normal behavior while the stress response is expected to decay towards normal behavior.

Insect walking is a complex behavior requiring precise control over 6 legs and interlimb coordination. Nevertheless, the precise motions underlying behavior can be characterized owing to its reproducibility and the ability to define leg trajectories. Previous studies indicated that insect walking is adaptive, as demonstrated by adaptive changes to walking slope or body load [12], [13]. Thus, insect walking behavior is suited for analyzing nuanced effects of stressors on motor function.

The cAMP pathway affects multiple forms of behavioral plasticity, such as learning [14]. The role of cAMP signaling in the stress response, however, is unknown. Studies in mammalian models have demonstrated changes in the behavioral response to stress in cAMP pathway mutants [15]–[18]. Similarly, *Drosophila* studies suggested that cAMP pathway mutants are hypersensitive to aversive stimuli which elicit stress [19], however a direct link to motor function has not been characterized.

Here, we quantified motor changes elicited by perturbations on components of *Drosophila* walking. We found that two independent stressors generated a common modification of leg trajectories. These stressors, electric shock and prolonged motion restriction, correspond to stress treatments demonstrated in mammals. Additional stressors facilitated the response, providing further evidence that independent stressors may converge to a common behavior. The response to both electric shock as well as suspension stress was amplified in adenylyl cyclase and synapsin mutants. Furthermore, the stress response was induced at a faster rate in these mutants. Our findings identify and dissect processes that underlie motor modifications in response to stress.

## Materials and Methods

### Fly stocks

Experiments in *Drosophila* were performed with 2 to 4 days old female flies. We received the following stocks from other groups: *Rut^1^* (Ronald Davis), and *Syn^97^* (Bertram Gerber). *Rut* and *Syn* mutants were backcrossed for six generations onto a wild type Canton S genetic background. The presence of the mutations in each generation was determined using allele specific PCRs.

### Video capture and analysis

Videos were obtained with a high-speed camera fitted with a custom zoom, focus and lighting (Bioimaging Solutions) at 100 fps. The first continuous, unidirectional stretch of walking was captured for analysis. The positions of the front edge of the head, the tip of the abdomen and the tips of all six legs were determined for each frame using ImageJ Manual Tracker. We analyzed positional data using a custom java script and performed a comprehensive analysis of locomotion parameters (see Table 1). For each time point, the script automatically ascertained whether a given leg was in stride, stance or whether the leg was retrogressing. Retrogression was defined as the ratio of backwards motion of the front legs to the total distance travelled by the fly. For amputated flies, retrogression was only defined for the amputated leg.

**Table 1:** Walking analysis parameters.

	Term	Description	Reference
1	Backtracking	Total distance that legs slip backwards divided by total distance travelled. Indicates efficiency of motion.	
2	Slipping Angle	Average angle of slipping for a given leg.	
3	Stride Period	Average time elapsed during a complete cycle of the stride and stance phases of motion.	[50]
4	Period Coefficient of Variation	Standard deviation of stride period divided by average of stride period. Indicates variability in motion timings.	
5	Stride Duration	Average time elapsed during the stride phase of motion.	[51]–[53]
6	Stance Duration	Average time elapsed during the stance phase of motion.	[51]–[53]
7	Interstep Lag	Average time elapsed between initiations of stride in a given pair of legs.	[50]
8	Interleg Stride Overlap (%)	Average percentage of time two given legs are simultaneously in stride.	[53]
9	Cross Correlation Coefficient	Similarity in the motion-time waveforms of two given legs. Describes regularity of motion in terms of each of 15 leg pairs.	
10	Leg-body distances	Average distances between the legs and the center of the body.	
11	Leg-leg distances	Average distances between the tips of two given legs.	
12	Stance Angle Deviation	Deviation in relative leg positioning from an equilateral triangle during stance. Minimal during normal motion.	
13	Anterior Extreme Position (AEP)	Touchdown distances of the fly legs, expressed in a body-centered coordinate system	[54]
14	Posterior Extreme Position (PEP)	Takeoff position of the fly legs, expressed in a body-centered coordinate system	[54]
15	AEP\PEP Variability	Vector sum of standard deviation of AEP or PEP positions. Indication of variability in foot positioning.	[53]
16	Step Amplitude	Average stride leg movement relative to body center, normalized to body length. Independent of speed.	[52]
17	Step Distance	Average stride length, in body lengths. Dependent on speed.	[50]
18	Step Distance Coefficient of Variation	Standard deviation of stride length divided by average of stride length. Indicates noise in stride lengths.	

The table lists the parameters that are calculated from the frame-by-frame acquisition of coordinates. Reference appears whenever a parameter has been used before.

### Electric Shock

**Figure 1:**
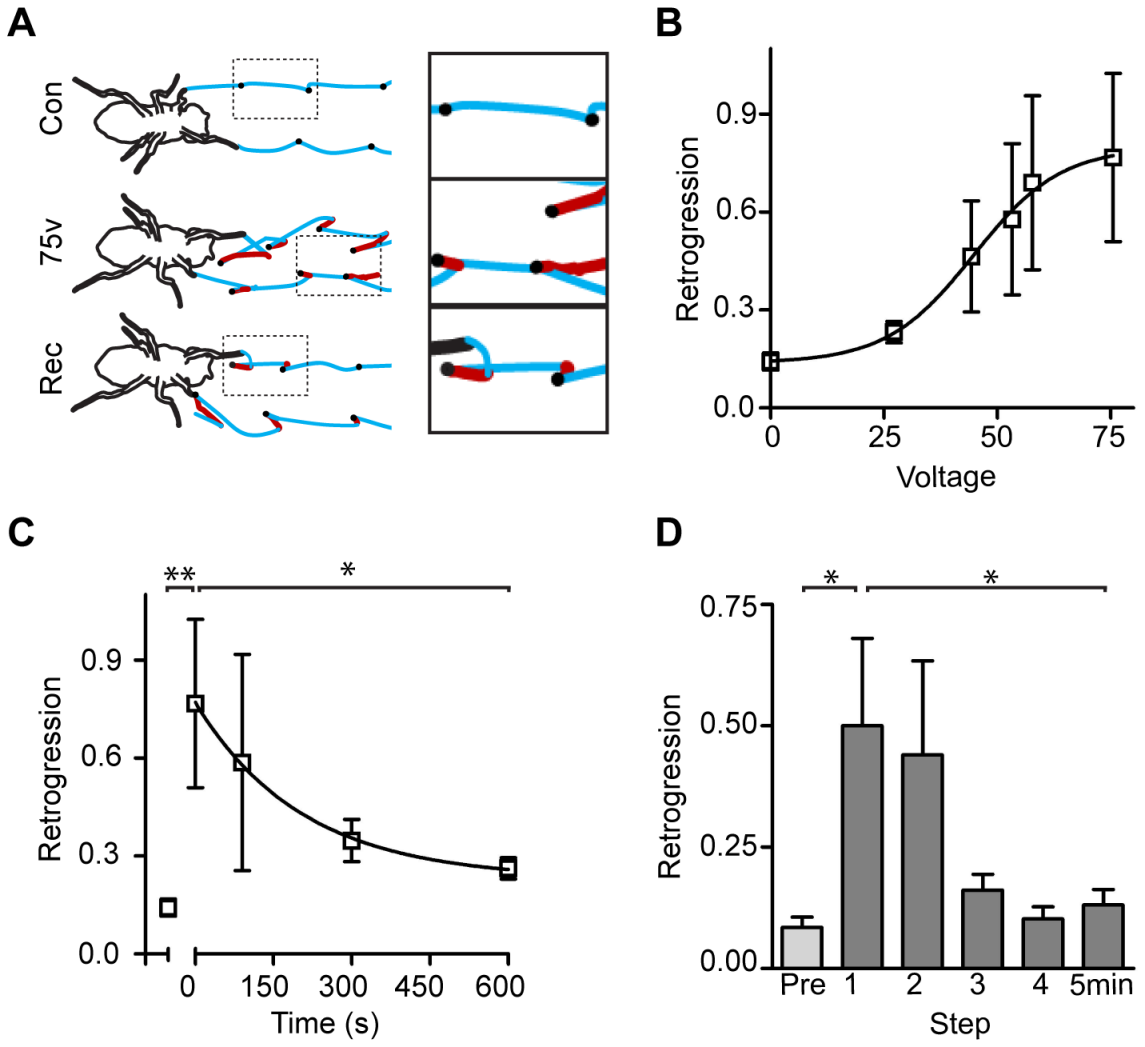
Effects of electric shock on fly walking. **A**. Trajectory of leg motions (blue lines) in control, unperturbed flies (Con). Black dots are touchdowns. During a 75V electric shock (75V), the legs retrogress (red line). During the recovery, following termination of the electric shock (Rec) retrogression gradually decreases. **B**. Intensity-response curve presenting retrogression as a function of applied voltage. Retrogression is defined as backwards motion of the forelegs normalized to total progression of the body. Data were fit to a boltzmann sigmoid. n = 12. The effect of voltage on retrogression was significant (p = 0.018, Kruskal-Wallis test). Post-hoc Dunn's multiple comparison test demonstrated significant difference (p<0.01) at 58 and 75 volts compared with 0 volts. **C**. Average retrogression before (left of breaker) and during (right of breaker) induction of a 2.5 hz, 75V stimulus. Note that retrogression habituates during the stimulus and that the steady-state of habituated level results in a substantial reduction of retrogression variability. Habituation was fit to a monoexponential decay. n = 12. p = 0.0013, Kruskal-Wallis test. *, p<0.05, Wilcoxon signed rank test. **D**. Retrogression lingers following the end of an electric shock. Note that the elevated retrogression recovers within 4 steps to pre-shock levels (black). p = 0.0071, Friedman test. *, p<0.05, Wilcoxon signed rank test with bonferroni-corrected p values for multiple comparisons. Error bars are mean ±SEM.

For electric shock experiments, flies were placed onto an electrified copper board (Fig. S1). The board was etched to form a grid pattern that ensured that the fly was always in contact with both ends and shorting the circuit. A fluon-coated plastic tube was placed onto the grid to prevent the fly from escaping the shock and the viewing area. We used a Grass SD9 stimulator to apply repeated 2.5 hz, 200 ms stimuli. All stimuli were at a 75 v setting except for the intensity response experiments in Fig. 1A. In order to study the combinatorial effects of slippery surfaces and electric shock, one to two day old female flies had their legs either amputated or covered in glue, and were then allowed to recover for a day in their home vials prior to experiments.

### Loss of traction

**Figure 2:**
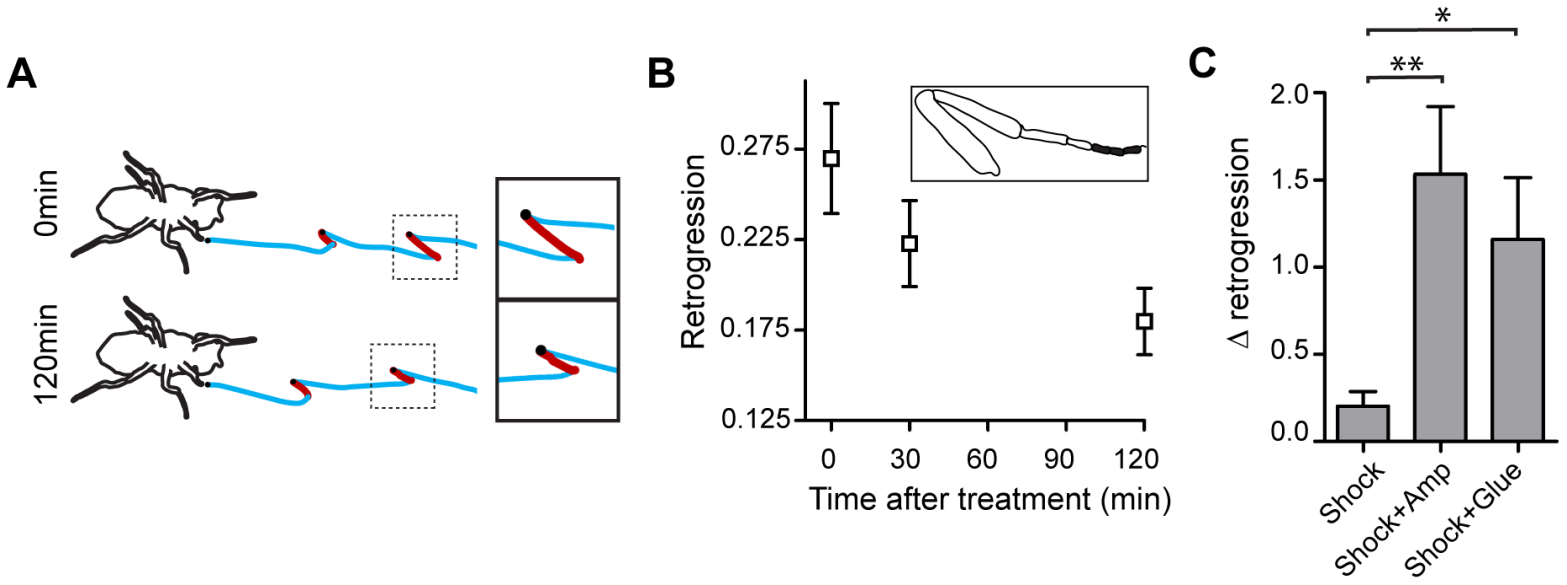
Effects of leg tip amputation on fly walking. **A**. Leg trajectories immediately after amputation of front left leg (0 min) and after 2 hours of recovery (120 min). Black dots are touchdowns. Blue lines represent forward motions in the amputated leg, while red lines represent retrogression. **B**. Retrogression at different times after amputation. Note that the retrogression following amputation (initial vs. final value) is not statistically significant. The inset is a schematic of the amputated front leg showing the amputated section in black shading. n = 17. **C**. Slippery surfaces amplify the effects of electric shock. Δ retrogression is the difference between retrogression during the first four steps of recovery from electric shock and retrogression prior to the electric shock, and controls for changes in baseline retrogression between the three conditions tested. n = 12–17. p = 0.0033, Kruskal-wallis test. * p<0.05, ** p<0.01, Mann-Whitney test with bonferroni correction of p values. Error bars are mean ±SEM.

All experiments that did not involve electric shock were performed in 2.5 cm dishes instead of electrified copper grids. In order to induce slipping, flies were amputated at the third tarsal subsection (Fig. 2B, inset). To cover the distal tarsal segment of the leg we used cyanoacrylate (superglue). Using an insect pin, we applied the superglue to the tips of the leg until the hook at the end of the fifth tarsomere was encapsulated in the material. In order to ensure that the superglue did not wear off, we examined all flies following the experiment to ensure that the superglue remained in place.

### Heat shock

For heat shock experiments, we submerged vials containing flies into a 37°C water bath and amputated the flies after 30 minutes of heating. Retrogression was observed at 0 minutes and 30 minutes of recovery.

### Suspension

In order to suspend the flies, we captured the flies by their wings using forceps. For amputated and suspended flies, flies were amputated prior to suspension. The forceps were then bound with clips in order to remain closed, and the flies were allowed to suspend for 2 hours. After the suspension period, flies were released and immediately captured on video.

To test the effects of leg-tip sensory feedback, the suspension assays were performed on flies that were allowed to contact the ground. One leg-tip was amputated in each fly to enhance the effects of motor adaptations. In order to determine the effect of stride feedback, we suspended flies while they were suspended over ¼ inch polypropylene balls. The balls were floating over a constant air supply, enabling the flies to walk freely.

### Statistics

Statistics were performed in Graphpad Prism 4 and all statistical tests are mentioned where they are used. Normality of data was tested using the Pearson omnibus normality test, and the appropriate tests were applied depending on whether the data were normally distributed or not.

## Results

To study the effects of stressors on motor function we have quantified the locomotor parameters of fly walking (Table 1) and analyzed the effects of the stressors on these parameters. We recorded walking using a high-speed camera, determined leg and body positions at each frame and derived data on leg trajectories. In preliminary tests of the assay, we found that stressors have a marked effect on the trajectory of legs. In normal walking, the forelegs move forward during the stride and then they are essentially lowered directly onto the surface (Movie S1). We found that when flies are challenged by stressors, they reach forward with their legs and then allow the legs to move backwards over the walking surface. We therefore used these retrogressions as readout of the motor changes induced by stress. Work in multiple species have determined that when insects are confronted with an obstacle they perform similar motions [20]–[23], which are expected to facilitate sampling of the terrain for a high-traction anchoring site. We reasoned that since this behavior represents a cautionary adaptation to adverse conditions, it may also constitute a general response to stressors.

To test whether stressed flies exhibit retrogression motions we applied an electric shock using an electrified copper grid (Fig. S1). Electric shock is commonly used as a stressor for similar studies in mammals [4], [24], [25]. Moreover, this stressor can be applied and withdrawn instantly and it does not elicit a mechanical disturbance to the execution of walking. We found that indeed, application of electric shocks resulted in retrogressions of the forelegs (Fig. 1A). To characterize the voltage dependence of the stress response we determined the intensity-response curve for retrogression, which fit a Boltzmann sigmoidal with a midpoint of 45±14v (Fig. 1B).

Studies in mammalian models as well as humans have demonstrated that animals rapidly habituate their stress response to repeated stimuli [26]–[28]. Habituation to repeated stress may represent a strategy of the nervous system to reduce its response to harmless perturbations [11]. In order to characterize whether the response to electric shock habituates, we applied repeated electric shocks and examined the response at varying time points during stimulation. We found that following the initial response to electric shock, retrogression decreased with a time constant of 206 seconds (Fig. 1C). Thus, as in other manifestations of stress, the retrogression response habituates during repeated electrical stimuli.

Stressors elicit behavioral changes that remain after withdrawal of the stimulus [5]. In order to test whether electric shock evokes lingering changes in motor performance, we examined motor performance after the end of an electric shock. We found that retrogression showed a trend towards higher values after electric shock termination which was statistically significant for the first step (Fig. 1D). Thus, the retrogression response persists as an aftereffect following the termination of stress application.

If the increase in retrogression following electric shock is a general response to stress, other stressors are expected to elicit a similar effect. Therefore, we tested whether the introduction of an independent stressor induces retrogression. A slippery walking surface introduces an unexpected and uncontrollable impediment to walking [29], [30], which may elicit stress. Accordingly, studies have shown that an unstable walking surface generated a behavioral stress response in mammalian models [31], [32], as well as in humans [33]. In order to create a slippery condition, we amputated the tip of the tarsus, thereby preventing the tarsal pad from adhering to the walking surface. The treatment caused the legs to slip backwards when flies were performing a stride, a motion that constitutes a baseline level of retrogression. This baseline level was determined at the end of a period of 2 hours of free walking post suspension. At the 0 min time point, retrogression showed a trend towards elevated levels relative to baseline, yet it was not statistically significant (Fig. 2A,B). The trend of retrogression to decline over tens of minutes probably reflects a process that resembles habituation, where each stride of the treated leg is effectively a repeated application of the stress. The tendency of amputated flies to display above-baseline retrogression suggests that a slippery surface evokes stress which is just below the significance threshold. Since exposure to one type of stressor may facilitate the subsequent response to a second stressor [34], we reasoned that combining electric shock with amputation should reveal the underlying effect of slippery surfaces on motor behavior. Indeed, the combination of electric shock and amputation induced substantial facilitation of the baseline retrogression induced by amputation alone (Fig. 2C, Fig. S2). Thus, slippery conditions are an independent physical driver of the retrogression stress response.

Since amputation is a harsh treatment, we sought an independent method for inducing loss of traction using a less damaging treatment. We induced retrogression by covering the last tarsal segment with a thin film of glue. As in the amputation experiment, covering the tarsal setae with glue facilitated the retrogression response to an electric shock (Fig. 2C). Facilitation of the response to electric shock following both amputation and glue treatment suggests that slippery condition drive the stress response in both cases. Accordingly, both amputation and glue treatment did not affect walking on high-traction surfaces such as paper (Retrogression on paper was 0.032±0.006 for control, 0.040±0.010 for amputated flies and 0.038±0.005 for glued flies.n≥7, p = 0.53 with Kruskal-Wallis test).

To further demonstrate the ability of independent stressors to facilitate the stress response we have combined amputation with heat shock, which is a known stressor [35]. As in the slippery conditions, heat shock was not sufficient for inducing robust retrogression (treated, 0.104±0.029 vs. untreated, 0.077±0.015, p = 0.75, Mann-Whitney test). However, the heat shock stress facilitated the response to amputation (amputation, 0.235±0.019 vs. heat shock with amputation, 0.319±0.034, p = 0.035, Mann-Whitney test). Thus, two stressors that do not elicit a response when applied separately give rise to a substantial stress response when applied sequentially. Taken together, our experiments combining stressors suggest that in addition to electric shock, slippery conditions and heat shock also drive the retrogression stress response.

**Figure 3:**
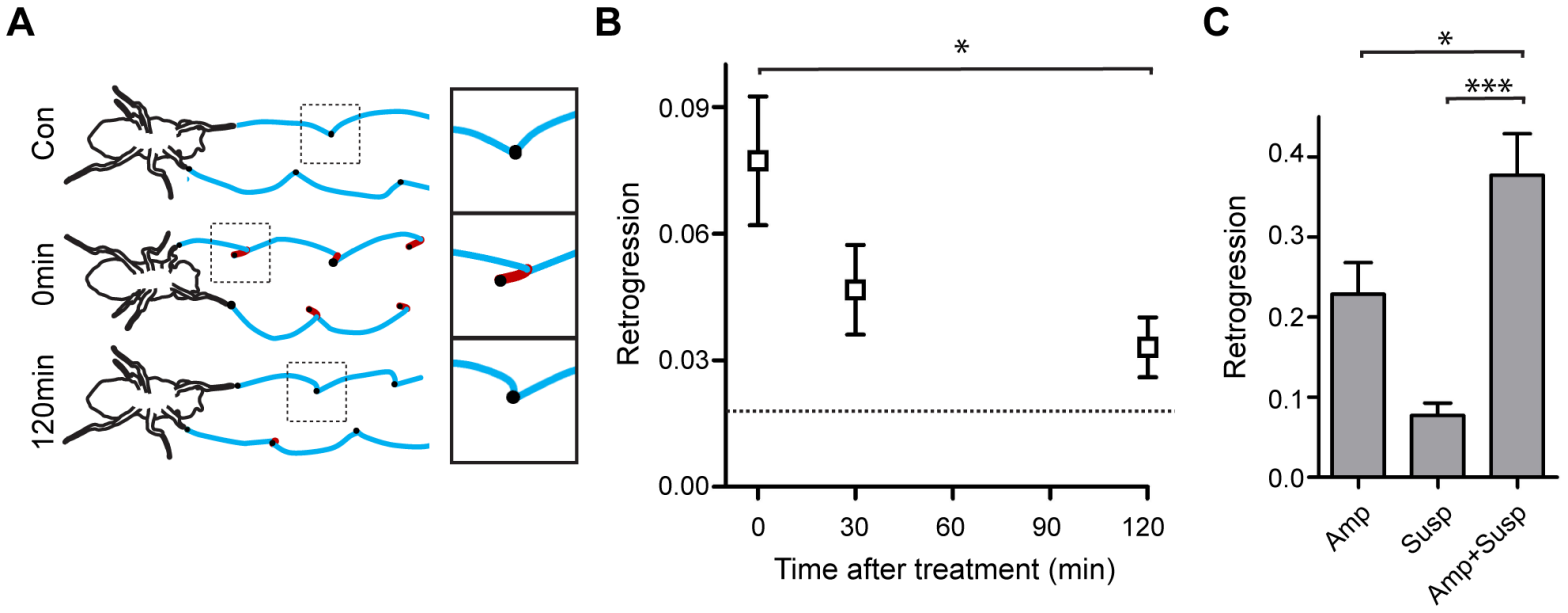
Effects of suspension on fly walking. **A**. Trajectory of leg motions (blue lines) in unperturbed flies (Con). Black dots are touchdowns. Immediately after suspension (“0 min”), the legs retrogress (red line). After 2 hours (120 min) retrogression is reduced. **B**. Backtracking at different times after release from suspension. Note that the initial retrogression gradually subsides. Dotted line represents retrogression in Con. n = 12. * p<0.05, One way repeated measures ANOVA with post-hoc Dunnett multiple comparison test. **C**. The effects of suspension and amputation are additive. The effect of suspension (Susp), amputation(Amp) and their combination (Susp+Amp) was defined as the difference between the initial (0 min) and the final (120 min) values. n = 12–17. p<0.0001, Kruskal-Wallis test. * p<0.05 *** p<0.001, Mann-Whitney test with bonferroni correction of p values. Error bars are mean ±SEM.

To validate that retrogression is indeed a response to stress, we sought to demonstrate the behavior using an independent, noninvasive assay. A commonly used method for inducing stress in rats is the tail-suspension test, whereby animals are stressed by suspending them in the air by their tail [36]. We adopted this method to the flies, suspending each fly in the air by its wings. Since it is impossible to study free locomotion during tethered suspension as we did during electric shock (Fig. 1A–C), we quantified the retrogression aftereffects following release of the flies (as in Fig. 1D). The behavior during tethered suspension appeared random, with alternating periods of agitated leg motions, grooming or remaining stationary. However, we found that after the end of a tethering period the flies demonstrated robust retrogressions that decayed over several minutes (Fig. 3A–B). The generation of retrogressions following a non-invasive treatment completely independent from electrical shock confirms that these motion modifications constitute a stress response.

**Figure 4:**
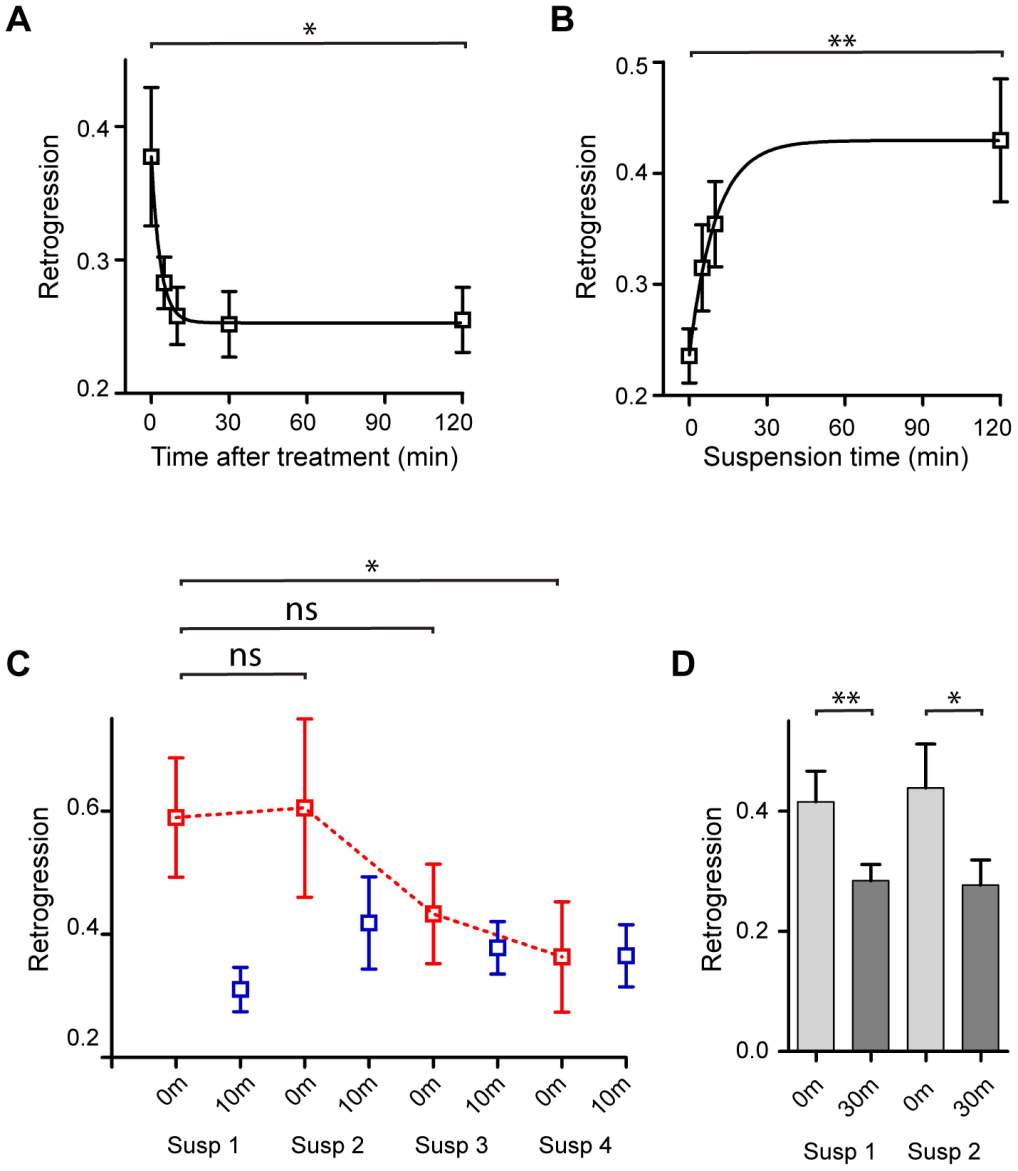
Effects of combining amputation and suspension on fly walking. **A**. Retrogression at different times after release from suspension. Amputated and suspended flies display a robust increase in backtracking that gradually decreases. Recovery of retrogression was fit to a monoexponential decay with a time constant of 11±2 min. n = 15. * p<0.05, Wilcoxon signed-rank test. **B**. Onset time course of walking adaptations. Flies were suspended for increasing durations and the retrogression was determined upon release at each time point. Data were fit to a monoexponential association with a time constant of 10 min. n≥10. p = 0.0025, Kruskal-Wallis test. * p<0.05 ** p<0.01, Mann-Whitney test with Bonferroni correction for multiple comparisons. **C**. Habituation of the response to combined amputation and suspension. The initial response upon release from suspension (blue) decreases to the baseline level of flies that have recovered from stress (red). p<0.05, Friedman test. * p<0.05, Wilcoxon signed rank test with bonferroni correction of p values. **D**. Sustained retrogressions could be repeatedly toggled. Retrogression at 0 min (red bars) and 30 min (blue bars) after two consecutive two hour suspension treatments (Susp1 and Susp2). n = 19–21. p = 0.0009, Friedman test. ** p<0.01 * p<0.05, Wilcoxon signed-rank test. Error bars are mean ±SEM.

As observed in previous experiments combining stressors, treatment with suspension and amputation resulted in facilitation of the stress response (Fig. 3C). This robust response, taken together with the slow recovery after suspension (Fig. 3B), allow for quantification of the kinetics of stress induction and recovery. The recovery rate of amputated and suspended flies was much slower than we observed in electrically shocked flies, taking minutes to plateau (compare Fg. 4A with Fig. 1D). Similar experiments in mammals demonstrated that induction of stress using motion restraint develops gradually [24]. To quantify the rate at which suspension induces the stress response we measured the initial retrogression of flies that had been suspended for different durations. This plot presents the accumulated perception of stress as a function of suspension time, as measured by the immediate retrogression aftereffect. Interestingly, the time-course of retrogression induction was similar to the recovery from this stress (Fig. 4B). These kinetic data suggest a single process drives both stress induction and recovery.

The kinetics of the stress response was different between suspension and electric shock, which is in accord with previous work in mammals that demonstrated variable aftereffect kinetics based on the type of stressor applied [25]. In accord with the slower induction and termination of the suspension stress response, we found that the habituation of retrogression also had a slower time course. Unlike electric shock, in which we observed habituation within a few minutes of a single session (Fig. 1A), suspension required multiple repeated sessions in order to habituate (Fig. 4C).

Reversibility of the response to stress would indicate that it results from a controlled behavior modification rather than from physical damage. To verify that the behavior change is reversible, we first tested the effect of suspension on flies with amputated leg tips, quantified retrogression and then repeated the process with the same flies. We found that a second suspension had an equivalent effect on walking parameters as the first (Fig. 4D). As in the changes following the first suspension, the effect of the second suspension decayed after 30 min. Thus, the retrogression that we observed following amputation was not a result of physical damage.

**Figure 5:**
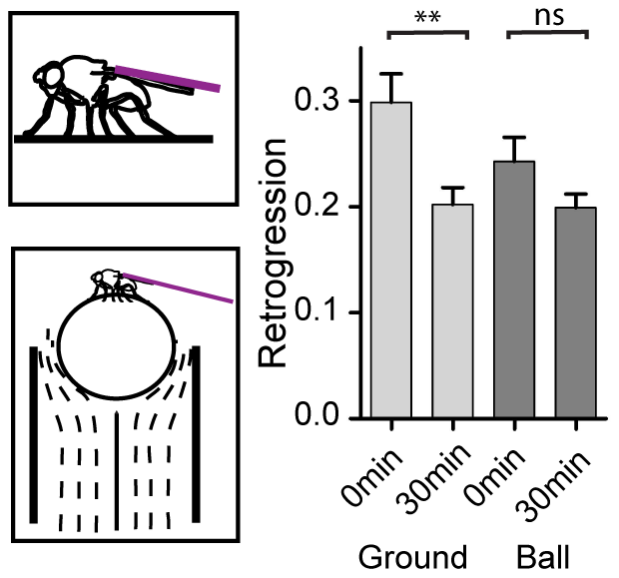
Effects of suspension method on the stress response. Top-left, schematic illustrates immobilization of fly by pin (purple line) while the fly is allowed contact with a flat surface. Bottom-left, schematic illustrates immobilization of fly by pin while fly is allowed to walk on a polypropylene ball suspended over a flow of air. Right, comparison of retrogression at 0 min and 30 min of recovery after 2 hours of immobilization with surface contact (red bars, top left schematic) or with treadball contact (blue bars, bottom left schematic). n = 23. ** p<0.01, Wilcoxon signed rank test. Error bars are mean ±SEM.

Work in mammals has demonstrated that the precise method of motion restraint affects the resulting behavioral response [27], [37], [38]. We reasoned that this response variability results from different perceptions of stress that were elicited by the sensory system. We therefore sought to dissect the sensory inputs that drive the retrogressions during tethered suspension. To test whether the loss of contact between the legs and the walking surface drives the stress response we tethered the flies at a normal walking distance over a surface, so that the flies could stand in the normal upright position (Fig. 5). We found that this suspension configuration resulted in a robust stress response that was comparable to the effect of suspension with no leg contact (Fig. 5). Therefore, the drive for retrogression does not arise from the loss of sensation of a walking surface. To test whether a perception of stress is generated by leg walking motions we suspended the flies over a ball that floats on an air stream (Fig. 5). Under this configuration, as the flies attempt to walk they rotate the ball backwards. Thus, although their body is immobilized, the legs perform normal walking and apply a propelling force that moves their body relative to the walking surface. In this configuration, the retrogression aftereffect showed a trend towards being increased, yet it was no longer significant (p = 0.15, Wilcoxon signed rank test). Taken together, these results indicate that the main drive for retrogression arises from sensing the relative motion between the legs and the walking surface. It is possible that the loss of absolute body motion drives the non-significant trend towards increased retrogression in treadball-mounted flies (Fig. 5).

**Figure 6:**
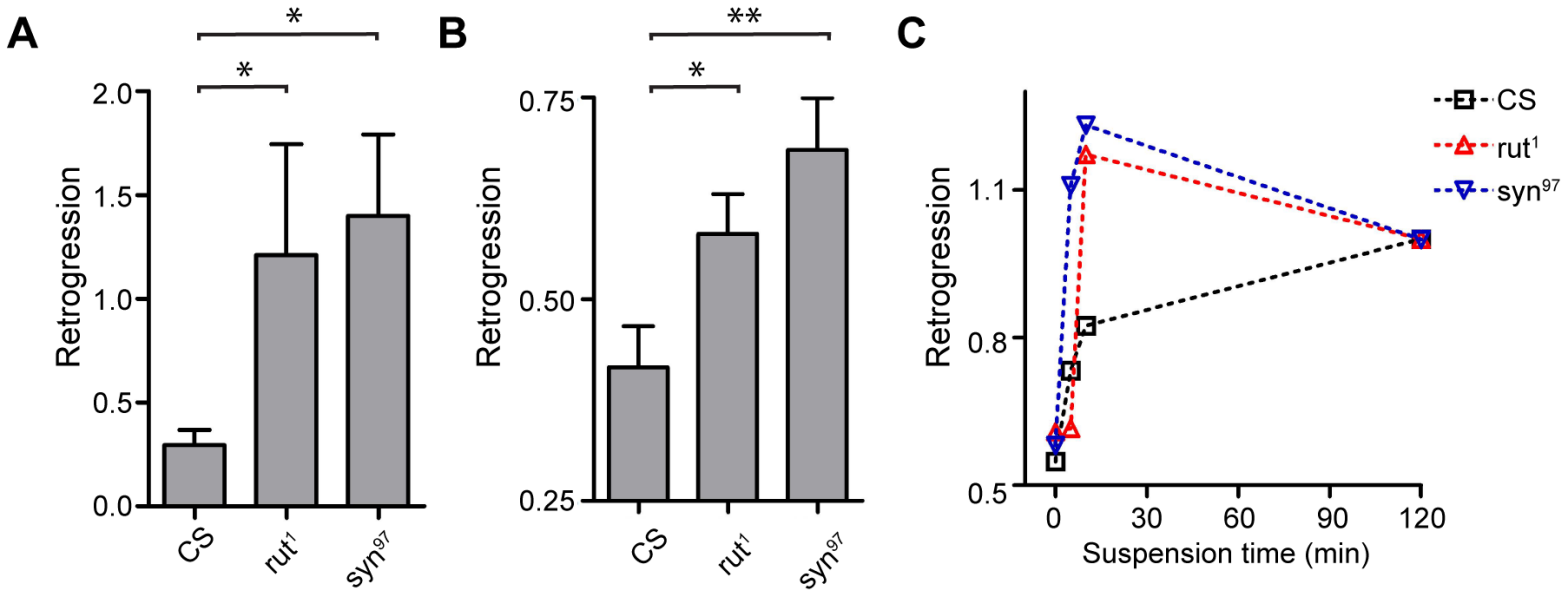
The effects of cAMP pathway mutants on retrogressions. **A**. cAMP pathway mutants demonstrate a stronger aftereffect following the end of electric shock. Retrogression was measured over the first four steps following an electric shock of 30 seconds. n = 16–20. p = 0.0097, Kruskal-Wallis test. * p<0.05, Mann-Whitney test with bonferroni correction for multiple comparisons. **B**. cAMP pathway mutants demonstrate a stronger aftereffect following the end of combined amputation and 2 hour suspension. n = 20–26. p = 0.0037, Kruskal-Wallis test. * p<0.05 ** p<0.01 Mann-Whitney test with bonferroni correction of p values. **C**. cAMP pathway mutants accumulate stress more rapidly than wild type flies. In order to compare the kinetics of stress induction between mutants, retrogression was determined immediately following different suspension times, and normalized to the retrogression levels after a 2 hour hanging period for each group. n≥8. Error bars are mean ±SEM.

Previous work in *Drosophila* and mammalian models suggests that components of the cAMP pathway modulate the behavioral response to stress [15]–[19]. Therefore, we sought to determine how mutants of the cAMP pathway affect the retrogression component of the response to stress. We examined mutations in the enzyme that generates cAMP, adenylyl cyclase, and in synapsin, which is a downstream effector [39]. To obtain uniform genetic background we backcrossed both mutants to the same wild type background for six generations. We observed an increased stress response in both mutants using both electric shock and suspension (Fig. 6A–B). Therefore, the cAMP pathway modulates the magnitude of individual stress responses. As a further dissection of the role of the cAMP pathway in the stress response, we took advantage of the slower rate of stress induction in the combined amputation and suspension assay (Fig. 4B). Interestingly, both mutants had a faster rate of stress induction than wild type (Fig. 6C). Taken together, our findings indicate that the cAMP pathway modulates both the intensity as well as the kinetics of the motor components of stress responses.

## Discussion

Our data show that exposure to a variety of stressors causes flies to perform backwards motions of their forelegs at the end of strides. These retrogressions during the stress response suggest that the default ‘panic mode’ of the fly is to adopt a safer posture and search for external anchor sites. A loss of contact between the legs and the walking surface results in reflexive searching motions [20], [21] that resemble the retrogressions that we observed. Thus, it is possible that the perception of stress engages existing reflex circuits to modify motor behavior. It is not clear if reflexive responses are components of the stress response in mammal stereotypies such as cage biting, head twirling and pacing, which do not serve any clear purpose [40]. Instead, the retrogressions may be most closely related to the instinctive drowning response [6], where people perform involuntary lateral motions of the arms that may be analogous to the insect searching motions.

In the experimental platform we developed, we analyzed the effects of stressors on motor function. The modification of fly walking by stressors exhibited several hallmarks of a response to stress. The most obvious characteristic that we observed was that the change in behavior was immediate, and decayed over time towards normal behavior. This is a characteristic of a behavioral stress response [3], and contrasts with the developing behavioral shift that is expected in motor learning. A second hallmark of a behavioral stress response is convergence of multiple stressors to a single response. We found that slippery surfaces, heat shock, suspension and electric shock all drove the retrogression stress response. Such convergence of distinct stressors onto a common response has been observed in mammals (e.g. a decreased time spent in the open arm of an elevated plus maze [41]), We also observed that the amplitude of the stress response varied between the different stressors. The level of response to each stressor might pertain to the risk level it presents to the animal's locomotion. For example, it is possible that heat treatment did not significantly affect retrogression by itself since ambient temperature does not directly impact locomotion. On the other extreme, electric shock constitutes a substantial stressing stimulus since it stimulates locomotion-relevant neurons in an unfamiliar and unexpected context. The third characteristic of the behavioral stress response is the ability of one stressor to facilitate the response to a different stressor (Fig. 2C, 3C). Facilitation has been observed in rodents in a variety of behavioral assays [34], [42]. Finally, we observed a decrease in retrogression when we repeatedly applied the same stressor, a process of habituation that has been observed for a wide range of stressors in mammals [11]. Repeated stimulation with electric shock resulted in robust habituation after a few minutes (Fig. 1C). Repeated stimulation by suspension generated significant habituation after four 10-minute cycles (Fig. 4C). Interestingly, while 40 minutes of intermittent suspension resulted in a robust habituation, 2 hours of continuous suspension resulted in a maximal stress response. These results suggest that repeated suspension generates habituation while the continuous treatment results in an accumulation of the stress response. Such an accumulation of stress response with increased restraint time is in agreement with mammalian studies [24]. In order to habituate the stress response to suspension, the fly needs repeated stimulation, which is in accord with the known features of habituation [11]. The similarities between *Drosophila* retrogressions and the mammalian stress responses suggest that common mechanisms link the two processes. Whether the similarities result from evolutionary conservation or from generation of analogous mechanisms, our experiments demonstrate that *Drosophila* is well suited as a model for studying the effects of stress on behavior.

The role of the immune system in the stress response may be an additional link between flies and mammalian models. The immune system is a component of the response to stress in vertebrates [43] and may play a role in the behavioral response to stress in invertebrates [44]. It is possible that our manipulations also evoke an immune response which plays a role in the behaviors that we observed. While the immediate effect of electric shock (Fig. 1) is probably emanating from direct modification of neuronal function, the prolonged treatments (such as heat shock or suspension) may have a component originating from the immune system.

Several characteristics of the stress response varied depending on whether flies were suspended or electrically shocked. The initial behavioral response, rate of retrogression induction, and rate of retrogression recovery all varied between the two stressors. Our results may be viewed in the context of similar comparisons that were made in mammals. Electrical footshock and immobilization/restraint stress are commonly compared with each other when studying the behavioral consequences of stress. Our observation that electrical shock induces a greater immediate behavioral response than immobilization is in agreement with previous studies that showed analogous results using a light-extinction test [4]. The varying rates of induction between the suspension assay and the electric shock assay were also similar to a previous study evaluating time spent in the light portion of a light\dark chamber. In that study, it was demonstrated that the effect of restraint stress required 30 minutes to saturate [24], which is similar to the kinetics that we observed using fly suspension. Additionally, as we observed in the flies, the effect of footshock rapidly saturated and did not require a long incubation in order to develop [24]. The last characteristic of the behavioral stress response is the rate of recovery, which occurred within a few steps following electrical shock and required several minutes following prolonged suspension. These results are similar to mammalian studies which have found a slower recovery to pre-stress levels following immobilization compared to electrical shock [25]. Thus, in both flies and mammals restraint elicits a stress response characterized by slow kinetics, while electrical stimulus elicits a rapid onset and decay of responses. It is possible that under natural settings such as a narrow opening or a sticky plant, restraint only represents a threat when prolonged, while the unfamiliar electrical stimulus evokes an immediate perception of stress.

In mammals, changing the method of immobilization alters the subsequent characteristics of the behavioral stress response [27], [37]. These findings suggest that sensory perception during stress induction determines the resulting motor output. Indeed, our experiments that modify the mode of suspension dissect which sensory input affects the stress response. Our findings indicate that contact with a walking surface does not mitigate the retrogression response. Relative motion between the legs and the walking surface, however, is a major factor in driving retrogressions. Interestingly, tethered locomotion above a treadball did not completely abolish the aftereffect (Fig. 5). Therefore, it is possible that the lack of absolute motion of the body relative to the environment also contributes to retrogressions. In immobilization experiments in mammals, in particular the tail suspension assay [36] which is most similar to our assay, the sensory input which underlies stress perception has not been dissected. Given the similarities we observed between retrogressions and mammalian stress responses, we expect that a main sensory input that drives stress in the tail suspension test is, as we observed, relative motion between the body and the walking surface.

The effect of cAMP on motor behavior has been shown in a wide range of animal models, ranging from aplysia [45] to mammals [46]. In order to define the role of the cAMP signaling pathway in generating a motor stress response, we examined the stress response in the mutants *rut^1^* and *syn^97^*. We found that in these cAMP pathway mutants, the motor stress response was enhanced (Fig. 6A–B). Accordingly, previous studies in the fly demonstrated that cAMP pathway mutants show decreased odor avoidance following electric shock [19]. Such studies are in accord with reports of mammalian adenylyl cyclase 5 knockout, where restraint stress causes animals to display increased anxiety [17]. Knockouts of mammalian adenylyl cyclase 8, however, actually show a decrease in anxious behavior in response to stress [15], [16], [18]. cAMP may influence neuronal function through activation of HCN channels [47], the EPAC exchange protein [48] and through activation of PKA [49] which phosphorylates downstream effectors such as Synapsin [39]. Since in our experiments the effect of synapsin ablation was similar to the effect of adenylyl cyclase mutation, it is possible that the main effect of cAMP signaling is through the adenylyl cyclase-cAMP-PKA-Synapsin pathway [39]. Such a scenario predicts that recruitment of synaptic vesicles at particular synapses is essential for curbing the stress response. It remains to be dissected which neruonal circuit mediates the effect.

## Supporting Information

Figure S1 **Schematic of grid used for electric shock experiments.** The fly is always in contact with the two sides of the circuit. The fly is confined to the camera viewing area by means of a fluon-coated plastic tube (dotted circle).
(TIF)

Figure S2 **The lingering effect of retrogression following electric shock on flies that had been pre-treated with leg amputation.** n≥8. p<0.0001, Kruskal-Wallis test. *** p<0.001 * p<0.05, Post-hoc Dunn's Multiple Comparison test. Error bars are SEM.
(TIF)

Movie S1 **The fly was placed in a plastic dish and suspended above the video camera.** The movie shows a ventral view of a walking fly exhibiting normal locomotion. Walking flies maintain a tripod of legs on the ground for support while the other tripod of legs stride forward (colored triangles). The alternation between tripods is analogous to human bipedal locomotion. The movie has been slowed 33-fold.
(MOV)

## References

[1] Shadmehr R, Brashers-Krug T (1997) Functional stages in the formation of human long-term motor memory. J Neurosci 17: 409–419. 8987766 10.1523/JNEUROSCI.17-01-00409.1997 PMC6793707

[2] McVea DA, Pearson KG (2007) Long-lasting, context-dependent modification of stepping in the cat after repeated stumbling-corrective responses. J Neurophysiol 97: 659–669. 17108090 10.1152/jn.00921.2006

[3] Koolhaas JM, Bartolomucci A, Buwalda B, de Boer SF, Flugge G, et al (2011) Stress revisited: a critical evaluation of the stress concept. Neurosci Biobehav Rev 35: 1291–1301. 21316391 10.1016/j.neubiorev.2011.02.003

[4] Mercier S, Frederic Canini, Buguet A, Cespuglio R, et al (2003) Behavioural changes after an acute stress: stressor and test types influences. Behav Brain Res 139: 167–175. 12642187 10.1016/s0166-4328(02)00265-6

[5] Min VA, Condron BG (2005) An assay of behavioral plasticity in Drosophila larvae. J Neurosci Methods 145: 63–72. 15922026 10.1016/j.jneumeth.2004.11.022 PMC2882685

[6] Vittone MaP, FM (2006) How to Recognize the Instinctive Drowning Response. On Scene: The Journal of US Coast Guard Search and Rescue P16100.4.

[7] Trompeter WP, Langkilde T (2011) Invader danger: lizards faced with novel predators exhibit an altered behavioral response to stress. Horm Behav 60: 152–158. 21549122 10.1016/j.yhbeh.2011.04.001

[8] Barreto RE, Barbosa-Junior A, Urbinati EC, Hoffmann A (2014) Cortisol influences the antipredator behavior induced by chemical alarm cues in the Frillfin goby. Horm Behav 65: 394–400. 24657662 10.1016/j.yhbeh.2014.03.007

[9] Padovan CM, Guimaraes FS (2000) Restraint-induced hypoactivity in an elevated plus-maze. Braz J Med Biol Res 33: 79–83. 10625878 10.1590/s0100-879x2000000100011

[10] Metz GA, Jadavji NM, Smith LK (2005) Modulation of motor function by stress: a novel concept of the effects of stress and corticosterone on behavior. Eur J Neurosci 22: 1190–1200. 16176362 10.1111/j.1460-9568.2005.04285.x

[11] Grissom N, Bhatnagar S (2009) Habituation to repeated stress: get used to it. Neurobiol Learn Mem 92: 215–224. 18667167 10.1016/j.nlm.2008.07.001 PMC2773683

[12] Grabowska M, Godlewska E, Schmidt J, Daun-Gruhn S (2012) Quadrupedal gaits in hexapod animals - inter-leg coordination in free-walking adult stick insects. Journal of Experimental Biology 215: 4255–4266. 22972892 10.1242/jeb.073643

[13] Keller BR, Duke ER, Amer AS, Zill SN (2007) Tuning posture to body load: decreases in load produce discrete sensory signals in the legs of freely standing cockroaches. J Comp Physiol A Neuroethol Sens Neural Behav Physiol 193: 881–891. 17541783 10.1007/s00359-007-0241-y

[14] Diegelmann S, Klagges B, Michels B, Schleyer M, Gerber B (2013) Maggot learning and Synapsin function. Journal of Experimental Biology 216: 939–951. 23447663 10.1242/jeb.076208

[15] Schaefer ML, Wong ST, Wozniak DF, Muglia LM, Liauw JA, et al (2000) Altered stress-induced anxiety in adenylyl cyclase type VIII-deficient mice. J Neurosci 20: 4809–4820. 10864938 10.1523/JNEUROSCI.20-13-04809.2000 PMC6772287

[16] Brewer JA, Bethin KE, Schaefer ML, Muglia LM, Vogt SK, et al (2003) Dissecting adrenal and behavioral responses to stress by targeted gene inactivation in mice. Stress 6: 121–125. 12775331 10.1080/1025389031000116460

[17] Kim KS, Han PL (2009) Mice lacking adenylyl cyclase-5 cope badly with repeated restraint stress. J Neurosci Res 87: 2983–2993. 19405150 10.1002/jnr.22119

[18] Razzoli M, Andreoli M, Maraia G, Di Francesco C, Arban R (2010) Functional role of Calcium-stimulated adenylyl cyclase 8 in adaptations to psychological stressors in the mouse: implications for mood disorders. Neuroscience 170: 429–440. 20638449 10.1016/j.neuroscience.2010.07.022

[19] Preat T (1998) Decreased odor avoidance after electric shock in Drosophila mutants biases learning and memory tests. J Neurosci 18: 8534–8538. 9763495 10.1523/JNEUROSCI.18-20-08534.1998 PMC6792831

[20] Durr V (2001) Stereotypic leg searching movements in the stick insect: Kinematic analysis, behavioural context and simulation. Journal of Experimental Biology 204: 1589–1604. 11398748 10.1242/jeb.204.9.1589

[21] Berg E, Buschges A, Schmidt J (2013) Single perturbations cause sustained changes in searching behavior in stick insects. Journal of Experimental Biology 216: 1064–1074. 23197090 10.1242/jeb.076406

[22] Pick S, Strauss R (2005) Goal-driven behavioral adaptations in gap-climbing Drosophila. Curr Biol 15: 1473–1478. 16111941 10.1016/j.cub.2005.07.022

[23] Pearson KGaFR (1984) Characteristics of leg movement and patterns of coordination in locust walking on rough terrain. Int J Robot Res 3: 101–112.

[24] MacNeil G, Sela Y, McIntosh J, Zacharko RM (1997) Anxiogenic behavior in the light-dark paradigm follwoing intraventricular administration of cholecystokinin-8S, restraint stress, or uncontrollable footshock in the CD-1 mouse. Pharmacol Biochem Behav 58: 737–746. 9329067 10.1016/s0091-3057(97)00037-3

[25] Marquez C, Belda X, Armario A (2002) Post-stress recovery of pituitary-adrenal hormones and glucose, but not the response during exposure to the stressor, is a marker of stress intensity in highly stressful situations. Brain Res 926: 181–185. 11814422 10.1016/s0006-8993(01)03112-2

[26] Wust S, Federenko IS, van Rossum EF, Koper JW, Hellhammer DH (2005) Habituation of cortisol responses to repeated psychosocial stress-further characterization and impact of genetic factors. Psychoneuroendocrinology 30: 199–211. 15471617 10.1016/j.psyneuen.2004.07.002

[27] Garcia A, Marti O, Valles A, Dal-Zotto S, Armario A (2000) Recovery of the hypothalamic-pituitary-adrenal response to stress. Effect of stress intensity, stress duration and previous stress exposure. Neuroendocrinology 72: 114–125. 10971146 10.1159/000054578

[28] De Boer SF, Koopmans SJ, Slangen JL, Van der Gugten J (1990) Plasma catecholamine, corticosterone and glucose responses to repeated stress in rats: effect of interstressor interval length. Physiol Behav 47: 1117–1124. 2395915 10.1016/0031-9384(90)90361-7

[29] Cham R, Redfern MS (2002) Changes in gait when anticipating slippery floors. Gait & Posture 15: 159–171. 11869910 10.1016/s0966-6362(01)00150-3

[30] Clark AJ, Higham TE (2011) Slipping, sliding and stability: locomotor strategies for overcoming low-friction surfaces. Journal of Experimental Biology 214: 1369–1378. 21430214 10.1242/jeb.051136

[31] Sarenac O, Lozic M, Drakulic S, Bajic D, Paton JF, et al (2011) Autonomic mechanisms underpinning the stress response in borderline hypertensive rats. Exp Physiol 96: 574–589. 21421701 10.1113/expphysiol.2010.055970 PMC3272224

[32] Hashiguchi H, Ye SH, Morris M, Alexander N (1997) Single and repeated environmental stress: effect on plasma oxytocin, corticosterone, catecholamines, and behavior. Physiol Behav 61: 731–736. 9145944 10.1016/s0031-9384(96)00527-6

[33] Carpenter MG, Frank JS, Adkin AL, Paton A, Allum JH (2004) Influence of postural anxiety on postural reactions to multi-directional surface rotations. J Neurophysiol 92: 3255–3265. 15295016 10.1152/jn.01139.2003

[34] Bhatnagar S, Vining C (2003) Facilitation of hypothalamic-pituitary-adrenal responses to novel stress following repeated social stress using the resident/intruder paradigm. Horm Behav 43: 158–165. 12614646 10.1016/s0018-506x(02)00011-9

[35] Patton ZJ, Krebs RA (2001) The effect of thermal stress on the mating behavior of three Drosophila species. Physiol Biochem Zool 74: 783–788. 11731970 10.1086/323327

[36] Steru L, Chermat R, Thierry B, Simon P (1985) The tail suspension test: a new method for screening antidepressants in mice. Psychopharmacology (Berl) 85: 367–370. 3923523 10.1007/BF00428203

[37] Klenerova V, Sida P, Krejci I, Hlinak Z, Hynie S (2007) Effects of two types of restraint stress on spontaneous behavior of Sprague-Dawley and Lewis rats. J Physiol Pharmacol 58: 83–94. 17440228

[38] Grissom N, Iyer V, Vining C, Bhatnagar S (2007) The physical context of previous stress exposure modifies hypothalamic-pituitary-adrenal responses to a subsequent homotypic stress. Horm Behav 51: 95–103. 17054953 10.1016/j.yhbeh.2006.08.011

[39] Michels B, Chen YC, Saumweber T, Mishra D, Tanimoto H, et al (2011) Cellular site and molecular mode of synapsin action in associative learning. Learn Mem 18: 332–344. 21518740 10.1101/lm.2101411

[40] Pomerantz O, Paukner A, Terkel J (2012) Some stereotypic behaviors in rhesus macaques (Macaca mulatta) are correlated with both perseveration and the ability to cope with acute stressors. Behav Brain Res 230: 274–280. 22366267 10.1016/j.bbr.2012.02.019 PMC3635133

[41] Walf AA, Frye CA (2007) The use of the elevated plus maze as an assay of anxiety-related behavior in rodents. Nat Protoc 2: 322–328. 17406592 10.1038/nprot.2007.44 PMC3623971

[42] Hauger RL, Lorang M, Irwin M, Aguilera G (1990) CRF receptor regulation and sensitization of ACTH responses to acute ether stress during chronic intermittent immobilization stress. Brain Res 532: 34–40. 2178035 10.1016/0006-8993(90)91738-3

[43] Elenkov IJ, Chrousos GP (2006) Stress system–organization, physiology and immunoregulation. Neuroimmunomodulation 13: 257–267. 17709947 10.1159/000104853

[44] Adamo SA (2012) The effects of the stress response on immune function in invertebrates: an evolutionary perspective on an ancient connection. Horm Behav 62: 324–330. 22381405 10.1016/j.yhbeh.2012.02.012

[45] Byrne JH, Baxter DA, Buonomano DV, Cleary LJ, Eskin A, et al (1991) Neural and molecular bases of nonassociative and associative learning in Aplysia. Ann N Y Acad Sci 627: 124–149. 1679307 10.1111/j.1749-6632.1991.tb25918.x

[46] Iwamoto T, Okumura S, Iwatsubo K, Kawabe J, Ohtsu K, et al (2003) Motor dysfunction in type 5 adenylyl cyclase-null mice. J Biol Chem 278: 16936–16940. 12665504 10.1074/jbc.C300075200

[47] Gonzalo-Gomez A, Turiegano E, Leon Y, Molina I, Torroja L, et al (2012) Ih current is necessary to maintain normal dopamine fluctuations and sleep consolidation in Drosophila. PLoS One 7: e36477. 22574167 10.1371/journal.pone.0036477 PMC3344876

[48] Shakiryanova D, Zettel GM, Gu T, Hewes RS, Levitan ES (2011) Synaptic neuropeptide release induced by octopamine without Ca2+ entry into the nerve terminal. Proc Natl Acad Sci U S A 108: 4477–4481. 21368121 10.1073/pnas.1017837108 PMC3060249

[49] Skoulakis EM, Kalderon D, Davis RL (1993) Preferential expression in mushroom bodies of the catalytic subunit of protein kinase A and its role in learning and memory. Neuron 11: 197–208. 8352940 10.1016/0896-6273(93)90178-t

[50] Strauss R, Heisenberg M (1990) Coordination of legs during straight walking and turning in Drosophila melanogaster. J Comp Physiol A 167: 403–412. 2121965 10.1007/BF00192575

[51] Graham D (1972) A behavioural analysis of the temporal organisation of walking movements in the 1st instar and adult stick insect (Carausius morosus). Journal of Comparative Physiology 81: 23–52.

[52] Wosnitza A, Bockemuhl T, Dubbert M, Scholz H, Buschges A (2013) Inter-leg coordination in the control of walking speed in Drosophila. J Exp Biol 216: 480–491. 23038731 10.1242/jeb.078139

[53] Mendes CS, Bartos I, Akay T, Marka S, Mann RS (2013) Quantification of gait parameters in freely walking wild type and sensory deprived Drosophila melanogaster. elife 2: e00231. 23326642 10.7554/eLife.00231 PMC3545443

[54] Cruse H (1976) The function of the legs in the free walking stick insect, Carausius morosus. Journal of Comparative Physiology 112: 235–262.

