# Supplementary figures and images for: The Effect of Stress on Motor Function in Drosophila
Source: PLoS One. 2014 Nov 6;9(11):e112076. doi: 10.1371/journal.pone.0112076 (PMC4222978; doi:10.1371/journal.pone.0112076)

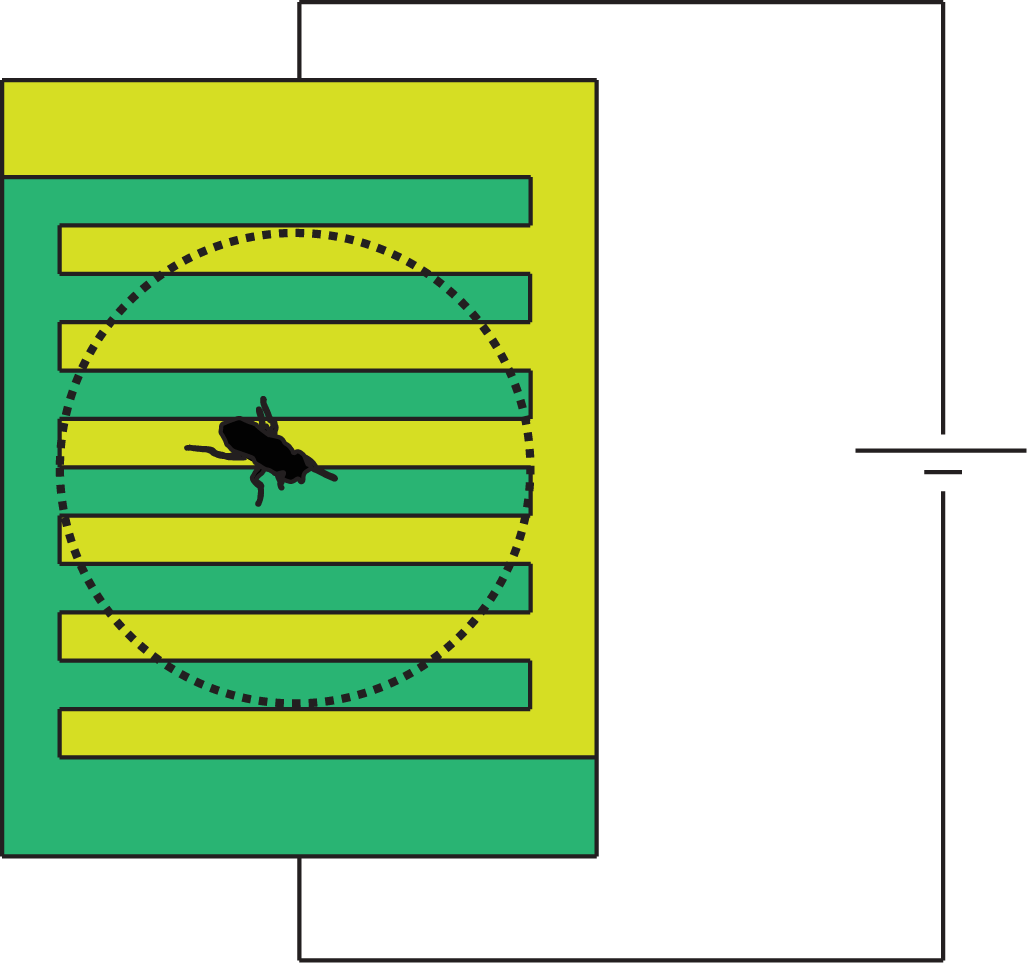

Supplement: Figure S1 — Schematic of grid used for electric shock experiments. The fly is always in contact with the two sides of the circuit. The fly is confined to the camera viewing area by means of a fluon-coated plastic tube (dotted circle). (TIF) [file pone.0112076.s001.tif]

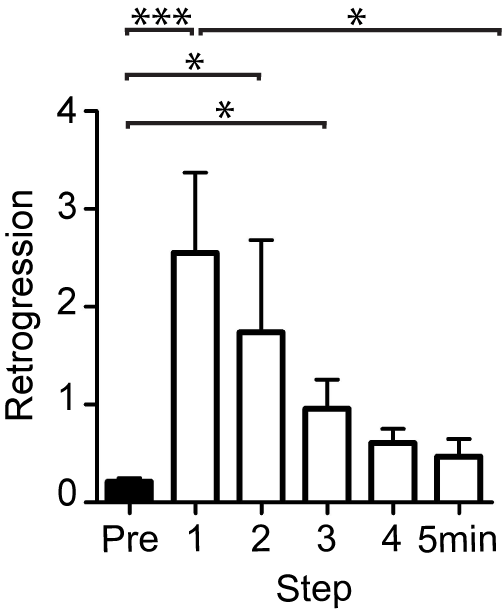

Supplement: Figure S2 — The lingering effect of retrogression following electric shock on flies that had been pre-treated with leg amputation. n≥8. p<0.0001, Kruskal-Wallis test. *** p<0.001 * p<0.05, Post-hoc Dunn's Multiple Comparison test. Error bars are SEM. (TIF) [file pone.0112076.s002.tif]
